# A diet change from dry food to beef induces reversible changes on the faecal microbiota in healthy, adult client-owned dogs

**DOI:** 10.1186/s12917-017-1073-9

**Published:** 2017-05-30

**Authors:** Kristin M. V. Herstad, Karina Gajardo, Anne Marie Bakke, Lars Moe, Jane Ludvigsen, Knut Rudi, Ida Rud, Monika Sekelja, Ellen Skancke

**Affiliations:** 1 Department of Companion Animal Clinical Sciences, Faculty of Veterinary Medicine, Norwegian University of Life Sciences (NMBU), Oslo, Norway; 2 Department of Basic Sciences and Aquatic Medicine, Faculty of Veterinary Medicine, Norwegian University of Life Sciences (NBMU), Oslo, Norway; 3 Faculty of Chemistry, Biotechnology and Food Science, Norwegian University of Life Sciences (NMBU), Ås, Norway; 4 Nofima, Norwegian Institute of Food, Fisheries and Aquaculture Research, Ås, Norway; 5 Department of Molecular Medicine, Institute of Basic Medical Sciences, Faculty of Medicine, University of Oslo, Oslo, Norway

**Keywords:** Client-owned dogs, Minced beef, Faecal microbiota, High throughput sequencing, Short chain fatty acids

## Abstract

**Background:**

Diet has a major influence on the composition of the gut microbiota, whose importance for gut health and overall well-being is increasingly recognized. Knowledge is limited regarding health implications, including effects on the faecal microbiota, of feeding a diet with high content of red meat to dogs, despite some owners’ apparent preference to do so. The aim of this study was to evaluate how a diet change from commercial dry food to one with a high content of boiled minced beef and vice versa influenced the faecal microbiota, and short chain fatty acid profile in healthy, adult, client-owned dogs.

**Results:**

The diet change influenced the faecal microbiota composition and diversity (Shannon diversity index). The most abundant OTUs in samples of dogs fed the dry food and high minced beef were affiliated with the species *Faecalibacterium prausnitzii* and *Clostridia hiranonis* respectively. The high minced beef diet apparently also influenced the short chain fatty acid profile, with increased isovaleric acid, as well as an increase in faecal pH. These effects were reversed when the commercial dry food was reintroduced in weeks 6 and 7.

**Conclusions:**

Results of this study can aid in the understanding of how diet changes influence the faecal microbiota and metabolite content on a short-term basis. Long-term studies are required to investigate potential implications for canine gut and general health.

**Electronic supplementary material:**

The online version of this article (doi:10.1186/s12917-017-1073-9) contains supplementary material, which is available to authorized users.

## Background

The canine faecal microbiota consists of a complex ecosystem of bacteria, virus, fungi and protozoa, of which bacteria dominate and are the most characterized organisms [1–4]. These bacteria are thought to heavily colonize the colon, and play a vital role in several functions in the host. Disruptions in the delicate balance of microorganisms has been associated with numerous maladies in humans, including inflammatory bowel disease [5, 6]. This has also been suggested to apply to dogs [7, 8].

Due to the ease of collection, faecal samples are commonly used to describe the intestinal bacteria, hence the term faecal microbiota. However, this reflects communities present in the distal part of the colon more closely than the more proximal parts of the intestine [9, 10].

The dietary content of macronutrients − carbohydrates, proteins and fat − can have a marked impact on the composition and function of the faecal microbiota, as shown in both dogs [11, 12] and in humans [13, 14]. Especially the fermentation of non-digestible carbohydrates by the colonic bacteria results in the formation of short chain fatty acids (SCFAs), mainly acetate, propionate and butyrate, and a lowering of the colonic pH [11, 15]. Data indicate that particularly butyrate is a preferred energy source for the colonocytes, and has in addition anti-inflammatory and anti-neoplastic properties [16–18], suggesting that butyrate is beneficial for gut health. In contrast, fermentation of proteins and amino acids by proteolytic bacteria in the colon results in increased faecal pH, and in the formation of faecal metabolites such as ammonia, sulphides, phenols, indols, and branched chain fatty acids (BCFA) including isovaleric acid. These may be harmful for gut health [11, 19–21]. Studies regarding the influence of dietary fat on the faecal microbiota are more scarce in both dogs and humans. However, high fat intake is associated with secretion of bile acids and these may alter the composition of the intestinal microbiota, as was reported in a study with rats [22]. Importantly, the proportion of one macronutrient to the total energy intake inherently influence the contribution from other macronutrients to the total energy intake. Thus, the effect of a change in one macronutrient on the faecal microbiota is therefore a result of the combinatory effect of all the macronutrients [23].

Knowledge of the canine faecal microbiome has lagged behind that of humans, but has recently improved with the implementation of state-of-the-art, high throughput sequencing methods (HTS). It is now evident that phylogenetic and metabolic similarities exist between dogs and humans [1]. Most studies examining diet-induced influences on canine faecal microbiota have evaluated effects of non-digestible carbohydrates [1, 11, 24, 25]. Three papers have reported the effects of animal-derived proteins, specifically greaves meal [12, 26, 27]. However, an overall picture of the bacterial community profile in response to the diet shift was not provided. Another study evaluated diet-induced shifts on the faecal bacterial community as an effect of raw beef and chicken, with or without yeast cell extract and inulin, using HTS methods [28]. However, that particular study focused on the effects of adding prebiotics and not the meat per se.

Dogs appear to have coevolved with humans, and have developed characteristics enabling them to efficiently digest a more carbohydrate-rich diet compared to their wild predecessor [29]. Yet fresh meat-based diets are common, due to some dog-owners’ and veterinarians’ belief that these diets are beneficial for dog health [30]. In humans, high consumption of red meat and reduced content of non-digestible carbohydrates in the diet have been associated with an elevated risk of inflammatory bowel disease and colorectal cancer, as reviewed by [31, 32]. It has been hypothesized that these associations are mediated through changes in colonic bacterial populations [33, 34]. Given that humans and dogs live in close contact and may have many microbes in common [1, 35], knowledge of the faecal microbiota in dogs, including potential zoonotic and pathogenic bacteria, may be of importance for both species [1].

To minimize variability among study subjects, diet-induced effects on the faecal microbiota have most commonly been investigated in laboratory dogs, most often beagle dogs in controlled environments [12, 24, 25, 28, 36]. Although these studies are highly valuable, the results are not necessarily applicable to a heterogeneous population consisting of client-owned dogs from various home locations.

To this end, more studies are needed regarding the consequences of feeding meat-based diets, including red meat, on the ecology of intestinal microbiota in non-laboratory dogs using more sensitive state-of-the-art methods. Data reported here are from a seven-week dietary intervention study designed to evaluate the effect of increasingly substituting a commercial dry food (CD) diet with boiled minced beef (MB) on the faecal microbiota composition using HTS in healthy, adult, client-owned dogs. The plasticity of the resident microbiota was assessed by reintroducing the CD diet following the MB diet periods.

## Methods

### Animals

Eleven healthy, client-owned dogs were recruited to participate in the seven-week (2 + 1 + 1 + 1 + 2) prospective dietary intervention study. Dog owners were employed at the Norwegian University of Life Sciences (NMBU) and included veterinarians and veterinary nurses. To be included, the dogs had to be clinically healthy with a normal haematological and serum biochemistry panel, no history of dietary intolerance, and no antibiotic treatments during the last 6 months prior to the study. Faeces were examined for parasites by standard methods at the Parasitology Laboratory, NMBU and included flotation/McMaster, sucrose flotation and immune fluorescence assay test (IFAT). All but one dog (dog no. 9) tested negatively for parasites. This dog tested positively for *Giardia* spp., and following treatment with fenbendazol (50 mg/kg for 5 days) and a subsequent negative test, this dog was included in the study. Detailed demographics of the 11 dogs are supplied in Table 1. Briefly, the dogs represented different breeds, were between 1.5 and 8 years of age, and the body weight was between 10 and 30 kgs. The dogs had been fed various types of commercial dry food diets, some also with small amounts of table scrapes. Only one of the 11 dogs regularly received a mixture of commercial dry food diet and a raw or boiled commercial meat-based diet. All dogs had normal body condition scores between 4 and 5 on a 9-point scale [37].

**Table 1:** Demographic overview of the 11 client-owned dogs included in a seven-week dietary intervention study

Dog no.^a^	Breed	Sex	Age (years)	Body weight (kg)
		Female F/Male M		
1	English Springer Spaniel	F	8	19.5
2	Mixed breed	F	3	15.4
3	Small Munsterlander	F	6	21.5
4	Eurasier	F	1.5	17.7
5	Irish Setter	M	4	21.5
6	Mixed breed	M	5	14.7
7	English Setter	M	5	28
8	English Cocker Spaniel	M	3	19
9	Mixed breed	F	6	28.7
10	English Cocker Spaniel	F	8	10.3
11	German Shorthaired Pointer	F	3	19.9

^a^Dog no. 2, 8 and 9 did not complete all the diet periods

### Study design and diets

All dogs followed the same diet regime adjusted to their individual estimated metabolizable energy (ME) requirements. The energy requirement for each adult dog was estimated according to information provided by the owner on type and amount of feed provided prior to initiation of the study and/or the range of 350–500 kJ ME x BW^0,75^ based on activity level, coat quality, body weight and body condition score [38]. During the first 2 weeks, the dogs were acclimated to a commercial dry food diet (CD1). The energy required to maintain a stable body weight during CD1 was used to calculate the rations provided during the subsequent feeding periods. After the CD1 period, dogs were fed a mixture of CD with increasing substitution of the CD with MB in three increments over a period of 3 weeks, 1 week on each MB-containing ration. The amount of MB given each week was calculated to provide 25 (low minced beef, LMB), 50 (moderate minced beef, MMB) and 75 (high minced beef, HMB) percent of the dogs’ total energy requirement. This resulted in increasing amounts of animal-derived fat and protein, with corresponding lower levels of carbohydrates and fibre in the rations (see Table 2). Following the 3 weeks on the MB-containing diets, dogs were again given the original CD diet without MB for 2 weeks (CD2). From each diet period, one freshly voided faecal sample was collected on each of the last three consecutive days, except for the last diet period CD2, in which one sample was collected from each of the last 2 days. Veterinary clinical examination, including registration of body weight and body condition score were performed every 7th day throughout the study. Blood samples for hematological and serum biochemical evaluation were collected at the start of the study and after completing the MB diets. Owners recorded appetite, faeces production and possible deviations from the feeding regime during the whole study period. An overview of the study design, including time schedule and sample collection is illustrated in Additional file 1: Table S1.

**Table 2:** Ingredients and nutrient composition of the rations during the seven-week dietary intervention study

	Rations				
	CD	LMB	MMB	HMB	MB
Ingredients, % of fresh weight in ration					
CD	100	61	34	15	-
MB	-	39	66	85	100
Nutrient composition, g/100 g DM					
Crude protein	27.1	32.5	38.9	46.2	55.3
Crude lipid	16.3	21.0	26.7	33.1	41.2
NFE	48.3	39.1	28.1	15.6	0
Crude fibre	1.2	1.0	0.7	0.4	0
Fibre (NSP)	10.4	8.4	6.1	3.4	0
Ash	7.0	6.4	5.6	4.7	3.5
ME (MJ/100 g DM)	1.80	1.93	2.09	2.28	2.50
DM in ration, as fed	92.2	69.5	53.8	42.7	34.0

*Abbreviations* and diet codes: *CD* commercial dry food (Felleskjøpet’s Labb adult), *DM* dry matter, *HMB* high minced beef, *LMB* low minced beef, *MB* minced beef (retail sourced, Norway), *ME* metabolizable energy, *MJ* megajoules, *MMB* moderate minced beef, *NFE* nitrogen-free extract, *NSP* non-starch polysaccharides

Ingredients in the CD diet, Labb adult (Felleskjøpet, Norway) are listed in Additional file 2: Table S2. The rations provided during each diet period and their nutrient compositions, including calculated content of the macronutrients (proteins, lipids, nitrogen-free extract and fibre) are provided in Table 2. The fresh MB, consisting of beef muscle and adipose tissue meant for human consumption (retail sourced, Norway) was packed and delivered to dog owners. The point of using MB was to provide a source of red meat for owners easily to portion, boil and mix with the CD diet. The owners were instructed to weigh the dry food and meat according to the feeding regime set up for each individual dog. Water was added to minced beef at a ratio of 3 parts MB:1 part water and simmered for 15 min or until the meat was completely cooked. The meat with any remaining water was mixed with the CD, cooled, and served. The reason for boiling the meat rather than serving it raw was to minimize the content of food-derived microbes. Owners were instructed to comply strictly with the ration plan and not feed their dogs other food-items, including snacks or supplements during the study period. The owners were also instructed to prevent their dogs from consuming non-food items such as garbage, faeces, grass and puddle water during the study period.

Solitary episodes of diarrhoea outside the sampling period were tolerated, provided the dog otherwise presented with good clinical health. Dogs with diarrhoea during the sampling period and/or had more than one single episode of diarrhoea were immediately taken off the MB-containing diet and moved on to the CD2 diet. To evaluate whether pathogenic bacteria caused the diarrhoea, both faecal samples and the raw and fed MB were analysed for the presence of coliform bacteria and *Salmonella* spp. However, faecal samples from the diet periods prior to the diarrhoea episodes, as well as from the CD2 period, were included in the study, provided a faecal consistency score within normal range was achieved.

### Faecal collection and sample storage

Owners were instructed in proper collection and handling of faecal samples. They collected samples from dogs during natural defecation, avoiding contamination from the ground. Samples were put directly in clean plastic bags. A representative sample was divided in three aliquots, kept in clean plastic containers and frozen within 2 hours. Samples were either aliquoted by the owner and frozen in the owners’ home freezers (−20 °C) or by the investigator in a centralized storage unit at the Norwegian University of Life Sciences (NMBU) at −80 °C. Samples stored at −20 °C in home freezers were transported on ice within a few weeks to the central −80 °C storage unit until further processing.

### Faecal consistency score, pH and water content

Owners registered faecal consistency daily, as well as episodes of diarrhoea or constipation. Diarrhoea was indicated with a faecal score from 4.5 to 5 according to the Waltham faeces scoring system [39]. The investigator also recorded faecal consistency score in all the collected samples. Faecal pH was measured by a portable pH meter with glass electrode (Knick Portamess 910) in a mixture of 1 g of faeces and 4 g of sterile water [27]. The average of three measurements for each sample was recorded for each dog and sampling time.

Faecal water content was recorded in samples from the last three consecutive days of each diet period. The water content was calculated from the difference in faecal weight of samples before and after freeze-drying to a constant weight (Christ Alpha 1–4; SciQuip, Shropshire, UK) [40].

### Short chain fatty acids

One faecal sample from each dog, taken the last day of each diet period, was used for the analysis of the SCFAs: acetate, butyrate, propionate and isovaleric acid by gas chromatography (GC). The method was based on [41, 42]. All chemicals were obtained from Sigma-Aldrich, Netherlands. As an internal standard, 2-ethyl butyric acid was added to PBS at a concentration of 2 μM. Faecal samples were thawed on ice, weighed and homogenized with the internal standard mix at a ratio of 1:3. Thereafter samples were centrifuged (17,000 × g, 10 min) and then filtered (0.22 μm diameter). Methanol containing 200 mM internal standard, was mixed with formic acid at a ratio of 6.4:1. This solvent was used to dilute filtered supernatant to a ratio of 50:50. Acetic, propionic, butyric and isovaleric acids were used as external standards at various concentrations in methanol. From each sample, 1 μl was injected into an Agilent GC HP-FFAP column (length 30 m, diameter 0.32 mm, film thickness 0.25 μm). The gas chromatography instrumentation Agilent 7890A was used, coupled with auto-sampler and flame ionisation detector (240 °C). The column was heated at a rate of 8 °C/min from 100 °C to 180 °C and 20 °C/min from 180 °C to 200 °C. Total running time was 17.5 min.

## Microbiota

### DNA extraction

From each dog, all faecal samples from each diet period were used for the sequencing analysis. Samples were thawed on ice and ~200 mg from each sample was added to sterile water at a ratio of 1:3 and homogenized. Microcentrifuge tubes containing 250 mg of glassbeads (size <106 μm; Sigma-Aldrich USA) were filled with S.T.A.R. (Stool Transport and Recovery; Roche, Basel, Switzerland) buffer solution and homogenized with 150 μl of sample suspension at a ratio of ~1 (sample) to 3 (S.T.A.R. buffer). Mechanical lysis of bacterial cells in samples was performed by homogenization using a MagNaLyser (Roche) twice at 6500 rpm for 20 s with 1 min cooling at 4 °C between runs. Thereafter, samples were centrifuged at 13000 rpm for 5 min. The resultant supernatants were transferred to a KingFisher 96-well plate and DNA was extracted using the Mag Mini LGC kit (LGC Genomics, UK) according to the manufacturer’s recommendations. Adequate concentration and quality of DNA in samples were ensured by Quanti-iT picoGreen dsDNA assay (Life Tecknologies, USA), using Qubit™ flourometer (Thermofisher).

### PCR amplification and library preparation

Polymerase chain reaction (PCR) was performed in order to amplify the V3-V4 region of the 16S rRNA gene. The primer pairs used were PRK314F:5′- CCTA CGGGRBGCASCAG-3′ and PRK806R: 5′-GGACTACYVGGGTATCTAAT-3′ [43]. The PCR contained a 25 μl mixture of 1 μl DNA, 0.2 uM of each primer, 1.25 U HotFirePol ^®^ DNA polymerase (Solis BioDyne, Estonia), 12.5 U HotFirePol ^®^ buffer B2 (Solis BioDyne, Estonia), 2.5 mM MgCl_2,_ 200 μM dNTPs and nuclease free water (nfw). The PCR cycles included initial denaturation at 95° for 15 min; 25 cycles of denaturing (95 °C for 30 s), annealing (50 °C for 30 s), elongation (72 °C for 45 s) with a final cycle at 72° for 7 min. Resulting amplicons were purified using Agencourt ampure beads (AMPure XP Beckman-Coulter, USA). A second PCR was performed to generate the libraries for sequencing. PRK primers modified to include Illumina adapters and unique combinations of primer indexes (Tru-seq LT) were added to each sample. The PCR reaction included similar reagents in similar amounts as used in the PCR for amplification, except from the different primers which were used. The initial denaturation at 95 °C for 15 min; 10 cycles of denaturing (95 °C for 30 s), annealing (50 °C for 1 min), elongation (72 °C for 45 s, and a final cycle at 72 °C for 7 min. The final PCR products were pooled in equal concentrations and again purified using AMPure XP before being quantified using PerfeCTa^®^ NGS library quantification kit for Illumina^®^ sequencing platforms (Quanta Biosciences™). Paired-end sequencing using Miseq Reagent Kit v3 (Illumina^®^) was performed on Illumina Miseq 200 with 15% Phix DNA spike in to ensure sequence diversity.

### Sequencing analysis

The resulting 300 bp paired-end reads were analysed following the Quantitative Insights Into Microbial Ecology (QIIME) pipeline [44]. The forward and reverse raw reads were joined using fastq-join algorithm [45]. Thereafter, sequences were stringently filtered using method fastq filter available in Usearch v7 script package with E_max =0.5. Singletons were discarded. The reads were subsequently clustered within a 97% similarity threshold into Operational Taxonomic Units (OTUs) using UPARSE pipeline [46], implemented in USEARCH 7 [47]. A representative sequence from each OTU was annotated using Greengenes v 13.8 reference sequences [48]. The annotation “other” used in the classification of bacterial taxa, indicates that the taxonomy could not be determined at lower phylogenetic level for that particular sequence. For each sample, 4000 randomly selected sequences were used for statistical analysis. The rarefaction analysis was performed using the command alpha_rarefaction.py within QIIME 1.8 [44]. Microbial diversity metrics, such as “observed species” and “Shannon diversity index” within each subject at a given time point (alpha diversity) were calculated. To quantify the differences between the dog’s diet-associated faecal microbiota (beta diversity), the distance metric, weighted UniFrac analysis was performed and visualized as a principal coordinate analysis (PCoA) plot through Primer PERMANOVA 7 [49].

The sequences of particular biological interest were further characterized at species level using Basic Local Alignment Search Tool (BLAST) [50], optimized for highly similar sequences (megablast) [51], to obtain classification to species level if identity score reached 97%. The sequences used for this search is listed in Additional file 3: Table S3.

### Statistical analysis

The mean profile for each dog in each diet period was calculated and used for statistical analysis of alpha- and beta diversity. The weighted (based on the presence and relative abundance of the different OTUs) UniFrac distance metric from QIIME was used as input file to Primer PERMANOVA 7 [49] in order to test for significant differences in bacterial communities at genus level in samples from the different diet periods. *P*-values were obtained using type III sums of squares with 999 unrestricted permutations of raw data. Data from all the MB diet periods (LMB, MMB, HMB) were compared with the CD1 and CD2 diets. Linear discriminant analysis (LDA) effect size (LEfSe) [52] was used to detect bacterial taxa at genus level in differential relative abundances in the different diet periods. Results from the following parameters: Shannon diversity index, observed species, faecal water, faecal consistency score, and short chain fatty acids were presented as medians and minimum and maximum ranges for each of the diet periods. Due to missing values from diet period HMB and CD2, statistical analysis did not include results from all 11 dogs. The statistical differences between these parameters were calculated using non-parametric Wilcoxon matched pairs signed rank test without correction for multiple comparison (Graph Pad Software, La Jolla, CA v.7). A *p*-value below 0.05 was considered statistically significant, and a *p*-value between 0.05 and 0.09 was considered a trend.

## Results

### Compliance and clinical and physiological effect of diets

According to the clinical examinations, results of haematological and serum biochemical analyses, and the dog-owners’ daily recordings, all dogs remained healthy throughout the study. Dogs consumed their rations with only minor deviations. The low incidence of food intake other than the provided diet, was equally distributed between the diet periods. Body weights were maintained with less than 3 % mean deviation during the periods with minced beef supplementation.

Isolated incidences of diarrhoea outside the sampling period were reported from dog-owners during the CD1 period (2 dogs), LMB period (1 dog), MMB period (1 dog) and the HMB period (3 dogs). However, all faecal samples analysed (see below) were of normal consistency (faecal score ranging from 2.5 to 3.5). Three dogs did not contribute with samples from all diet periods due to a faecal score > 4.5 two consecutive times. One dog (no. 8) was taken off the MMB diet and another during the HMB diet (no. 2). The faecal consistency improved immediately when these dogs were reintroduced to the CD diet (CD2). The third dog (no. 9) did not complete the CD2 period. The presence of coliform bacteria and *Salmonella* spp. were below detection limits in the diarrhoeic samples, as well as in the raw and boiled (fed) MB.

### Faecal water, faecal consistency score, pH and SCFAs

Besides the isolated incidences of diarrhoea in some dogs outside the sampling period reported above, the medians for faecal water and faecal consistency score did not change throughout the study period (Table 3.). Faecal pH appeared to increase with the MB-supplementation, and was significantly different between the CD1 and the HMB periods (*p* = 0.02). A similar trend was observed when comparing the CD2 and HMB periods (*p* = 0.06; Table 3). Relative amounts of the SCFAs: acetic, propionic, butyric and isovaleric acids in the faecal samples are shown in Table 4. The HMB diet appeared to increase the relative amount of isovaleric acid compared to the CD1 and CD2 periods (*p* = 0.05 and *p* = 0.02, respectively), and of butyric acid compared to the CD2 period (*p* = 0.01). Higher relative amounts of acetic acid were observed in samples from the CD2 vs. HMB period (*p* = 0.01).

**Table 3:** Median♦ faecal pH, water, consistency, diversity index and observed species from the dietary intervention study

	Diet periods					Signed-Ranks test	
						*p*-values	
	CD1	LMB	MMB	HMB	CD2	CD1 vs. HMB^1^	CD2 vs. HMB^1^
pH^2^	6.51 [6.22–7.07]	6.55 [6.2–6.77]	6.67 [6.46–6.91]	6.72 [6.66–7.03]	6.49 [6.03–6.83]	0.016*	0.063**
Water (%)	46 [39–64.6]	45.2 [40.3–67.6]	46.6 [40.8–62.6]	46.6 [40.5–68.6]	50.22 [40.3–68.6]	0.7	0.9
Consistency score	2.5 [2.2–3]	2,9 [2–3]	2.5 [2.3–3.5]	2.5 [2.5–3]	2.6 [2–3]	>0.9	0.6
Shannon diversity index	4.4 [3.38–5.06]	4.42 [3.76–4.85]	4.36 [3.09–4.7]	4.27 [3.15–4.76]	4.49 [3.22–4.72]	0.03*	0.08**
Observed species	73 [49–102]	74 [48–90]	77 [48–98]	79 [46–104]	78 [50–90]	0.57	0.55

♦Maximum and minimum values are provided in brackets.

*Abbreviations* and explanation: The diet periods were as follows: CD1 for week 1 and 2, during which all dogs were acclimated to commercial dry food (CD; Felleskjøpet’s Labb adult), followed by incremental substitution of the CD diet with minced beef − *LMB* low minced beef for week 3, *MMB* moderate minced beef for week 4, and *HMB* high minced beef for week 5 – and finally, CD2 for week 6 and 7, during which the dogs were reintroduced to the CD diet.

^1^Wilcoxon-matched sign rank test without correction for multiple comparisons. *P*-value for CD1 vs. HMB was determined for 9 dogs and *P*-value for CD2 vs. HMB was determined for 8 dogs.

^2^P–values for faecal pH was determined for seven dogs (CD1 vs. HMB) and five dogs (CD2 vs. HMB), due to missing values.

*Considered statistically significant; ** Considered a trend

**Table 4:** Median♦ faecal short chain fatty acids (relative amounts) from the seven-week dietary intervention study

	Diet periods					Signed-Ranks test	
						(*p*-values)^1^	
	CD1	LMB	MMB	HMB	CD2	CD1 vs.HMB	CD2 vs.HMB
Acetic acid	53.2 [50.8–58.3]	52.9 [49.4–57.1]	52.5 [48.5–59.2]	52.0 [48.2–52.3]	55.4 [50.5–56.9]	0.4	0.01*
Butyric acid	11.1 [8–13]	11.1 [7.9–15.4]	11.0 [9.0–12.7]	10.9 [9.2–13.2]	10.5 [7.0–12.5]	0.5	0.01*
Propionic acid	32.8 [29.4–37]	32.9 [26.6–38.9]	33.1 [26.2–37]	32.7 [28–35.2]	32.5 [29.5–36.4]	0.6	0.7
Isovaleric acid	3.6 [1.3–4.4]	3.3 [1.3–5.6]	3.5 [2.2–4.1]	3.9 [1.7–5.9]	3.0 [1.6–4.0]	0.05*	0.02*

♦Maximum and minimum values are provided in brackets.

Abbreviations and explanation: The diet periods were as follows: CD1 for week 1 and 2, during which all dogs were acclimated to commercial dry food (CD; Felleskjøpet’s Labb adult), followed by incremental substitution of the CD diet with minced beef − LMB, low minced beef for week 3, MMB, moderate minced beef for week 4, and HMB, high minced beef for week 5 – and finally, CD2 for week 6 and 7, during which the dogs were reintroduced to the CD diet.

^1^Wilcoxon-matched sign rank test without correction for multiple comparisons. *P*-value for CD1 vs. HMB was determined for 9 dogs and *P*-value for CD2 vs. HMB was determined for 8 dogs.

*Considered statistically significant

### Sequencing analysis

Of the initial 139 faecal samples, five were discarded due to low sequencing depth. Processing of data resulted in a total of 5, 289, 167 sequences, on average 31, 297 per sample. The alpha diversity metric “observed species” curve reached a plateau with a mean of 75 observed species, indicating adequate sequencing depth (Additional file 4: Figure S1).

### Faecal microbiota

**Fig. 1:**
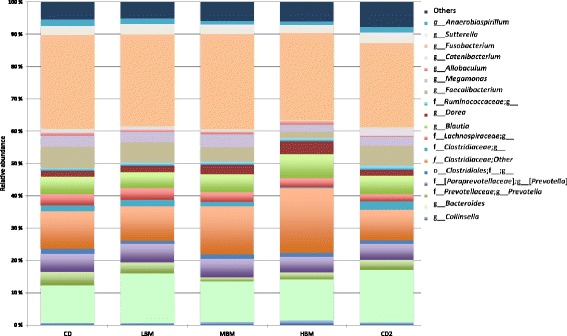
Mean relative abundances of the 15 most common operational taxonomic units (OTUs) at genus level. Data are from faecal samples taken following different diet periods from 11 healthy, client-owned dogs during the seven-week dietary intervention study. The diet periods were as follows: CD1 for week 1 and 2, during which all dogs were acclimated to commercial dry food (CD; Felleskjøpet’s Labb adult), followed by incremental substitution of the CD diet with minced beef − LMB, low minced beef for week 3, MMB, moderate minced beef for week 4, and HMB, high minced beef for week 5 – and finally, CD2 for week 6 and 7, during which the dogs were reintroduced to the CD diet

The most abundant bacterial phyla in samples of dogs were *Firmicutes* (43%), *Fusobacteria* (28%) and *Bacteroidetes* (22%), whereas *Proteobacteria* (5%) and *Actinobacteria* (1%) were less commonly observed. Mean relative abundances of the 15 most abundant genera in samples from each of the diet periods are depicted in Fig. 1, showing that *Fusobacterium* (28%), *Bacteroides* (14%) and *Clostridiaceae* other (14%) where the most dominant in all dogs.

**Fig. 2:**
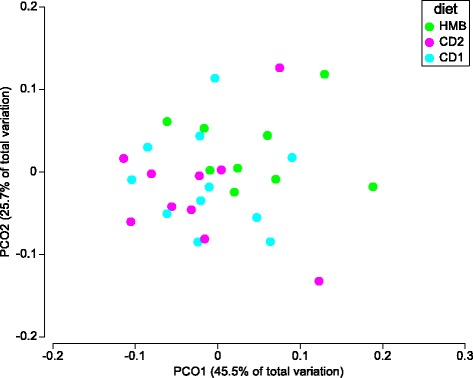
Principal Coordinate analysis (PCoA) on weighted UniFrac distance metric from QIIME using Primer PERMANOVA. Data are from faecal samples taken following different diet periods from 11 healthy client-owned dogs, during the seven-week dietary intervention study. A mean value of the three samples collected from each of the dogs in each of the diet periods were used for this analysis. The diet periods were as follows: CD1 for week 1 and 2, during which all dogs were acclimated to commercial dry food (CD; Felleskjøpet’s Labb adult), followed by incremental substitution of the CD diet with minced beef − LMB, low minced beef for week 3, MMB, moderate minced beef for week 4, and HMB, high minced beef for week 5 – and finally, CD2 for week 6 and 7, during which the dogs were reintroduced to the CD diet. The data are displayed across the two main principal coordinates (PCO 1 and 2). Each point represents the total bacterial community within one sample and each colour represents different diet period. Closer clustering between points indicate higher relative commonality with respect to bacterial community (more bacterial taxa in common). Concomitantly, larger distances between points indicate lower relative commonality in bacterial taxa. The different coloured points represent individual samples from dogs fed the different diets. CD1 (turquoise points), CD2 (purple points), HMB (green points). PERMANOVA for HMB vs. CD1, *p* = 0.04, *t* = 1.57 and HMB vs. CD2, *p* = 0.04, *t* = 1.61. No significant differences were detected between CD1 or CD2 vs. LMB or MMB and are therefore not included in the figure

**Fig. 3:**
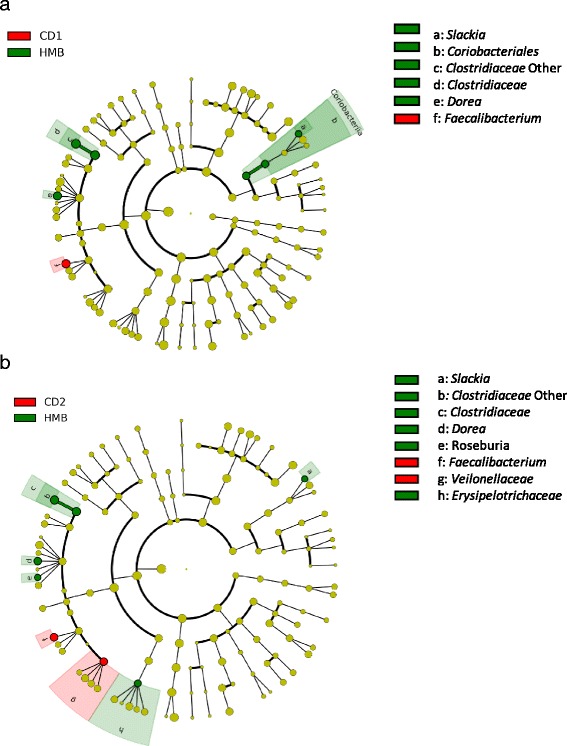
Circular cladogram representation of linear discriminant analysis effect size, LEfSe, of the 16S rRNA gene sequences. The sequences were obtained from faecal samples taken following different diet periods from 11 healthy, client-owned dogs during the seven-week dietary intervention study. A mean value of the three samples collected from each of the dogs in each of the diet periods were used for this analysis. The diet periods were as follows: CD1 for week 1 and 2, during which all dogs were acclimated to commercial dry food (CD; Felleskjøpet’s Labb adult), followed by incremental substitution of the CD diet with minced beef − LMB, low minced beef for week 3, MMB, moderate minced beef for week 4, and HMB, high minced beef for week 5 – and finally, CD2 for week 6 and 7, during which the dogs were reintroduced to the CD diet. The data points represent OTUs identified at phylum level in the centre of the circle (name not given), and genus level in the outer circle. The OTUs present in differential relative abundances in samples from the diet periods − red: CD1 and CD2, and green: HMB − are listed in the upper right corner. The yellow points indicate OTUs that are not present in differential relative abundances in samples from diet periods. Figure (**a**) depicts data from CD1 vs. HMB, and (**b**) depicts data from CD2 vs. HMB and (α = 0.05, LDA score > 2.0). *P*-value for CD1 vs. HMB was determined for 9 dogs and *P*-value for CD2 vs. HMB was determined for 8 dogs. No significant differences were detected between CD1 or CD2 vs. LMB or MMB and are therefore not included in the figures

Species richness and evenness assessed by Shannon diversity index were decreased in the HMB samples, compared with samples from the CD1 and CD2 periods (*p*-value 0.03 and 0.08, respectively) (Table 3). However, observed species was not significantly different between samples from dogs fed the different diets (Table 3). As visualized by a PCoA plot using the weighted UniFrac distance metric, the HMB samples clustered differently compared with samples from both the CD1 and CD2 diet periods, and these differences were significant (PERMANOVA, CD1 vs. HMB, *p* = 0.04, *t* = 1.57 and CD2 vs. HMB, *p* = 0.04, *t* = 1.61) (Fig. 2). There was no clear clustering of samples when comparing LMB vs. CD1/CD2 and MBM vs. CD1/CD2, suggesting that the macronutrients between these diets were too similar to influence the microbiota composition, or that the faecal microbiota required more time to adjust following initiation of the MB supplementation. Therefore, the following results only include the comparison between diet periods HMB vs. CD1 and HMB vs. CD2. To determine the OTUs present in differential relative abundances in the diet periods (CD1 vs. HMB and CD2 vs. HMB), LEfSe was used. The bacterial taxa *Clostridiaceae*, *Clostridiaceae* other, *Dorea*, *Coriobacteriales*, *Coriobacteriaceae,* and *Slackia* were more abundant in samples from dogs fed the HMB diet, whereas *Faecalibacterium* was more abundant in samples from dogs during the CD1 period (*p* < 0.05; LDA score > 2; Fig. 3a). Comparing HMB and CD2 periods, the abundance of *Clostridiaceae*, *Clostridiacea* other, *Dorea*, *Slackia*, *Erysipelotrichaceae* and *Roseburia* were increased in the HMB samples, whereas *Faecalibacterium* and *Veillonellaceae* were increased in samples from the CD2 period. (*p* < 0.05; LDA score > 2; Fig. 3b). A BLAST search was performed of the nucleotide sequence from OTU_2, classified as Clostridiaceae other, which was identified as *Clostridia hiranonis* with 97% identity*.* A BLAST search was also performed of the sequence classified from OTU_5 classified as *Faecalibacterium,* which was identified as *Faecalibacterium prausnitzii* with 98% identity (Additional file 3: Table S3).

## Discussion

This study investigated how the canine faecal microbiota, pH, and SCFA profile were influenced by a diet change from commercial dry food (CD) to a diet increasingly supplemented with minced beef (MB). These parameters were also assessed when the dogs were reintroduced to the CD diet. Although previous studies have demonstrated diet-induced effects on the canine faecal microbiota [12, 25], these have not shown whether effects are reversible.

The HMB diet apparently induced short-term changes in the faecal microbiota, with lower species diversity and changes in the genus level composition, which were reversed when dogs were reintroduced to the CD diet. A human dietary intervention study also demonstrated restoration of the microbiota when reverted to the original diet [13]. This indicates plasticity of the microbiota, since the microbiota adapts depending on the available diet substrate [13, 53]. Importantly, this study evaluated the effects of substituting the different nutrients/components in the CD diet with those in MB, while keeping constant energy intake. Although the following discussion focuses on the effects of increasing animal derived protein and fat, and decreasing contents of non-digestible carbohydrates, other diet components in these rations may also have had a role in shaping the faecal microbiota.

The HMB diet-related reduction in Shannon diversity index, which measures species richness and evenness, was not accompanied by a reduction in observed species, a measure of species richness. This indicates that the decrease in species diversity in HMB samples may be a result of a change in the proportion of species present (evenness), rather than presence or absence of various species (richness). In any case, the reduced species diversity, and possibly also the lower relative abundance of a bacterial taxa that was classified with 98% identity by a BLAST search as *Faecalibacterium prausnitzii*, may be a result of the low content of fibre in the HMB diet compared with the CD diet. High dietary levels of various types of non-digestible carbohydrates have been shown to increase faecal microbial diversity [54, 55]. On the other hand, the higher fat content in the HMB diet with a presumed concomitant increased secretion of bile acids that have antibacterial effects may also be a factor [56, 57]. Absence of *F. prausnitzii* in the faecal microbiota has been associated with inflammatory bowel disease in both humans [58] and dogs [7]. This may be due to decreased levels of the anti-inflammatory metabolite butyrate, which is efficiently produced by this bacteria [59]. However, the relative amount of butyrate was elevated in the HMB samples compared with the CD2 samples, possibly explained by the higher relative abundance of *Roseburia* in HMB vs. CD2 samples. This genus is also a known butyrate producer [60]. However, butyrate levels and *Roseburia* abundance did not significantly differ between CD1 and HMB samples. The content of dietary fibre has been associated with increased concentrations of dogs’ faecal SCFAs, including butyrate in one study [11], although a similar association has not been observed in other studies [61, 62]. An in-vitro study using faecal samples from cheetahs demonstrated that cartilage entering the large intestine may have a similar effect on the SCFA profile as plant fibre [63]. However, dogs in the present study received boiled minced beef consisting primarily of muscle and adipose tissue presumed to have a low content of cartilage. Whether the microbiota of dogs, irrespective of dietary fibre type and content, retain functional redundancy and produce adequate levels of butyrate to maintain a healthy gut, requires further investigations.

As mentioned above, another possible explanation for the reduced faecal microbiota diversity associated with the HMB samples, might be the antibacterial effect of an increased bile acid secretion in response to a lipid-rich diet [22, 64]. Specifically, the shift in fat content may explain the higher relative abundance of a bacterial taxa within the family *Clostridiaceae* in the HMB samples compared with the CD1 and CD2 samples. A BLAST search was used to classify this bacterium as *Clostridia hiranonis* with 97% identity. This bacterium is capable of dehydroxylating primary bile acids into secondary bile acids [65], which are considered to have carcinogenic potential [66]. A high fat diet in humans induce proliferation of bacteria with this ability [34]. *C. hiranonis* has so far been described as a normal commensal bacterium in faecal microbiota of healthy dogs [28, 67]. The higher proportions of *Coriobacteriales* in HMB samples vs. CD1 samples and the *Erysipelotrichaceae* in HMB samples vs. CD2 samples, may also be explained by the high fat content in the diet, as described in a study of hamsters and mice [68, 69]. Added insight into the clinical health implications of *C. hiranonis* and varying levels of the primary and secondary bile acids in dogs may be provided by correlating the abundance of *C. hiranonis* and *baiCD*, the microbial gene that encodes the 7a- dehydroxylating enzyme, using quantitative PCR in faecal samples of dogs fed low vs. high fat diets [34].

Due to dogs’ carnivorous origin, it is reasonable to assume that their faecal microbiota harbour proteolytic bacteria. This may explain the high relative abundance of the genus *Fusobacterium*, as corroborated by data from previous HTS studies in dogs [3, 25]. However, the HMB diet did not seem to change the relative abundance of this genus. In humans, *Fusobacterium* spp*.* has been implicated in the development of colorectal cancer [70, 71] and ulcerative colitis [72], diseases not commonly diagnosed in dogs [73, 74]. The relative abundance of another genus with proteolytic characteristics, *Bacteroides*, did not increase in faecal samples from dogs fed HMB. This contradicts research on humans, where both short- and long-term studies have shown higher proportion of *Bacteroides* in faecal microbiota after the consumption of a “Western diet” rich in animal protein and fat, and low in fibre [13, 14, 34, 54, 75]. The diverse outcome of diet-induced effects on faecal microbiota in different mammals should be considered in the light of evolutionarily or genetically defined resident bacteria present at the outset [76]. During human evolution, a major diet shift from a predominantly plant-based to an omnivorous diet occurred [77], whereas dogs developed from carnivorous predecessors and have adapted to utilizing a considerable amount of dietary carbohydrates during domestication [29], which may explain differences in the plasticity of the microbiota. This study investigated how a diet shift induces short-term changes on the faecal microbiota. Whether a long-term change in diet would lead to a similar and permanent shift in the microbial community merits further investigation. In any case, the brevity of the current study must be taken into consideration when interpreting the results of this study.

Both the MMB and HMB diets (protein content 39 and 46 g/100 g diet DM, respectively) led to loose faeces in some dogs, and caused recurrent diarrhoea in two dogs. Diarrhoea was also observed in dogs fed high level animal-derived protein (greaves meal; >50 g protein/100 g diet DM) according to previous studies [26, 61]. Diet-induced effect on faecal consistency has been associated with an increase in *Clostridium perfringens* in faecal samples [27] as well as ileal chyme [26] in laboratory dogs. The faecal samples analysed in our study had normal consistency and water content. The observed diarrhoeic episodes occurred outside the sampling periods. The influence of diarrhoea is therefore not directly reflected in our data, and might explain why *Clostridium perfringens* was not significantly increased by the HMB diet. Anyhow, the increased amount of isovaleric acid and pH in faecal samples of dogs fed HMB compared to CD1 and CD2, indicates that undigested proteins may reach the colon in at least some dogs consuming higher levels of proteins [20], and the proteolytic activities of bacteria may lead to increased levels of potentially detrimental metabolites. The implications for dog health are currently not known.

The advantage of having dog owners consisting mostly of veterinarians and veterinary nurses, who are highly aware of the importance of adhering to a study protocol, was to achieve higher compliance. However, some deviations from the study protocol cannot be completely ruled out, for instance accidental or unsupervised intake of other diets/non-food items than the prescribed diet, which could influence the faecal microbiota. Despite all this, our study revealed diet-induced changes in the faecal microbiota using a population of client-owned dogs. Using laboratory dogs instead, and thus evaluating diet-induced changes on a more homogeneous faecal microbial profile, may have resulted in less variation and hence even clearer results. However, the purpose with our study was to clarify how a diet change would induce effects, despite the various environmental factors also influencing the faecal microbiota.

HTS methods have opened up new opportunities to explore the complex and interactive community of microorganism present in the gut [10, 78, 79] or in faeces [1, 4, 25, 80, 81]. However, the different methods being used, such as the methods to lyse bacterial walls and generate libraries [82, 83], sequencing methods, and importantly the bioinformatics tools, have to be taken into account when comparing results between studies, as reviewed by [84]. In particular, the methods for clustering OTUs and the different databases used for annotation will at least partially explain variability. For this study, the UPARSE pipeline was used, which claims to improve biological accuracy of the OTUs, thus potentially lowering the number of observed OTUs [46]. This may have an impact on the low number of observed species in the dataset (median of 75 species per sample) compared to other studies with dogs (mean > 100 per sample) [7, 85]. Limitations with the use of HTS include difficulties in detecting bacterial taxa of low abundances, possibly affected by the diet shifts.

Future studies should include increased application of qPCR to determine specific bacteria that are influenced by the dietary content of red meat and non-digestible carbohydrates, which may play a role in modulating intestinal health. These would include sulphide reducing bacteria [13], mucin degrading bacteria [33], and butyrate-producing bacteria [86]. Additionally, elucidating functional properties of the faecal microbiota, including a broader spectrum of the metabolites they produce, might show even clearer differences caused by diet shifts [13]. Finally, some data indicate differing microbiota profiles when comparing faecal samples with intestinal mucosal samples [9]. Investigating bacteria in direct contact with the intestinal mucosa might be more relevant for studying bacteria related to gut health [87], but was not performed in these healthy client-owned dogs due to financial and ethical constraints.

## Conclusion

In a heterogeneous population consisting of 11 healthy client-owned dogs, exposure to a HMB diet seemed to induce changes in the faecal microbiota composition and decreased diversity, compared with the pre-exposure period when dogs were fed the CD diet. OTUs affiliated with the species *Clostridia hiranonis* were increased, whereas OTUs affiliated with the species *Faecalibacterium prausnitzii*, were reduced in the HMB samples. In addition, faecal pH increased and the levels of SCFAs were influenced, most notably by higher relative amounts of isovaleric acid in the HMB samples. Apparently, these changes were largely reversed when dogs were reintroduced to the original CD diet. Whether the diet-induced changes observed here have any implications for gut health in the long-term, needs to be evaluated in studies with larger number of animals performed over a longer period of time, and should include methods measuring a larger number of functional properties of the microbiota, such as metabolomics.

## Additional files

Additional file 1: Table S1. Study design, time schedule and sampling during the seven-week dietary intervention study. (DOCX 13 kb)

Additional file 2: Table S2. Main ingredients in Labb Adult commercial dry food diet (CD). Contents of ingredients are listed in falling order of proportion. (XLSX 9 kb)

Additional file 3: Table S3. Assigned taxonomy using BLAST of sequences derived from 16S rRNA gene sequencing. (XLSX 8 kb)

Additional file 4: Figure S1. Rarefaction analysis of V3-V4 16S rRNA gene sequences. The rarefaction curve shows observed species in samples of dogs fed different diets. (DOCX 100 kb)

Additional file 5: Table S4. Relative abundance of genera in the different diet periods. (L6 table generated with QIIME). (XLSX 46 kb)

## Abbreviations

16S rRNA: 16S Ribosomal RNA
bp: BasePair
dNTP: Deoxy nucleotide Triphosphate
PBS: Phosphate buffered saline

## References

[1] Swanson KS, Dowd SE, Suchodolski JS, Middelbos IS, Vester BM, Barry KA (2011). Phylogenetic and gene-centric metagenomics of the canine intestinal microbiome reveals similarities with humans and mice. ISME J.

[2] Handl S, Dowd SE, Garcia-Mazcorro JF, Steiner JM, Suchodolski JS (2011). Massive parallel 16S rRNA gene pyrosequencing reveals highly diverse fecal bacterial and fungal communities in healthy dogs and cats. FEMS Microbiol Ecol.

[3] Hand D, Wallis C, Colyer A, Penn CW (2013). Pyrosequencing the canine faecal microbiota: breadth and depth of biodiversity. PLoS One.

[4] Garcia-Mazcorro JF, Dowd SE, Poulsen J, Steiner JM, Suchodolski JS (2012). Abundance and short-term temporal variability of fecal microbiota in healthy dogs. Microbiol Open.

[5] Sokol H, Seksik P, Rigottier-Gois L, Lay C, Lepage P, Podglajen I (2006). Specificities of the fecal microbiota in inflammatory bowel disease. Inflamm Bowel Dis.

[6] Manichanh C, Rigottier-Gois L, Bonnaud E, Gloux K, Pelletier E, Frangeul L (2006). Reduced diversity of faecal microbiota in Crohn's disease revealed by a metagenomic approach. Gut.

[7] Minamoto Y, Otoni CC, Steelman SM, Buyukleblebici O, Steiner JM, Jergens AE (2015). Alteration of the fecal microbiota and serum metabolite profiles in dogs with idiopathic inflammatory bowel disease. Gut Microbes.

[8] Suchodolski JS, Markel ME, Garcia-Mazcorro JF, Unterer S, Heilmann RM, Dowd SE (2012). The fecal microbiome in dogs with acute diarrhea and idiopathic inflammatory bowel disease. PLoS One.

[9] Eckburg PB, Bik EM, Bernstein CN, Purdom E, Dethlefsen L, Sargent M (2005). Diversity of the human intestinal microbial flora. Science.

[10] Suchodolski JS, Camacho J, Steiner JM (2008). Analysis of bacterial diversity in the canine duodenum, jejunum, ileum, and colon by comparative 16S rRNA gene analysis. FEMS Microbiol Ecol.

[11] Simpson JM, Martineau B, Jones WE, Ballam JM, Mackie RI (2002). Characterization of fecal bacterial populations in canines: effects of age, breed and dietary fiber. Microb Ecol.

[12] Hang I, Rinttila T, Zentek J, Kettunen A, Alaja S, Apajalahti J (2012). Effect of high contents of dietary animal-derived protein or carbohydrates on canine faecal microbiota. BMC Vet Res.

[13] David LA, Maurice CF, Carmody RN, Gootenberg DB, Button JE, Wolfe BE (2014). Diet rapidly and reproducibly alters the human gut microbiome. Nature.

[14] Wu GD, Chen J, Hoffmann C, Bittinger K, Chen YY, Keilbaugh SA (2011). Linking long-term dietary patterns with gut microbial enterotypes. Science.

[15] Zimmer J, Lange B, Frick JS, Sauer H, Zimmermann K, Schwiertz A (2012). A vegan or vegetarian diet substantially alters the human colonic faecal microbiota. Eur J Clin Nutr.

[16] Macfarlane GT, Macfarlane S (2012). Bacteria, colonic fermentation, and gastrointestinal health. J AOAC Int.

[17] Goncalves P, Martel F (2013). Butyrate and colorectal cancer: the role of butyrate transport. Curr Drug Metab.

[18] Apanavicius CJ, Powell KL, Vester BM, Karr-Lilienthal LK, Pope LL, Fastinger ND (2007). Fructan supplementation and infection affect food intake, fever, and epithelial sloughing from Salmonella challenge in weanling puppies. J Nutr.

[19] Propst EL, Flickinger EA, Bauer LL, Merchen NR, Fahey GC (2003). A dose-response experiment evaluating the effects of oligofructose and inulin on nutrient digestibility, stool quality, and fecal protein catabolites in healthy adult dogs. J Anim Sci.

[20] Nery J, Goudez R, Biourge V, Tournier C, Leray V, Martin L (2012). Influence of dietary protein content and source on colonic fermentative activity in dogs differing in body size and digestive tolerance. J Anim Sci.

[21] Elsden SR, Hilton MG (1978). Volatile acid production from threonine, valine, leucine and isoleucine by clostridia. Arch Microbiol.

[22] Islam KB, Fukiya S, Hagio M, Fujii N, Ishizuka S, Ooka T (2011). Bile acid is a host factor that regulates the composition of the cecal microbiota in rats. Gastroenterology.

[23] Hewson-Hughes AK, Hewson-Hughes VL, Colyer A, Miller AT, McGrane SJ, Hall SR (2013). Geometric analysis of macronutrient selection in breeds of the domestic dog, *Canis lupus familiaris*. Behav Ecol.

[24] Vanhoutte T, Huys G, De Brandt E, Fahey GC, Swings J (2005). Molecular monitoring and characterization of the faecal microbiota of healthy dogs during fructan supplementation. FEMS Microbiol Lett.

[25] Middelbos IS, Vester Boler BM, Qu A, White BA, Swanson KS, Fahey GC (2010). Phylogenetic characterization of fecal microbial communities of dogs fed diets with or without supplemental dietary fiber using 454 pyrosequencing. PLoS One.

[26] Zentek J (1995). Influence of diet composition on microbial activity. I. Effects on varying protein intake on the composition of the ileum chyme and faeces. J Anim Physiol Anim Nutr (Berl).

[27] Zentek J, Fricke S, Hewicker-Trautwein M, Ehinger B, Amtsberg G, Baums C (2004). Dietary protein source and manufacturing processes affect macronutrient digestibility, fecal consistency, and presence of fecal *Clostridium perfringens* in adult dogs. J Nutr.

[28] Beloshapka AN, Dowd SE, Suchodolski JS, Steiner JM, Duclos L, Swanson KS (2013). Fecal microbial communities of healthy adult dogs fed raw meat-based diets with or without inulin or yeast cell wall extracts as assessed by 454 pyrosequencing. FEMS Microbiol Ecol.

[29] Axelsson E, Ratnakumar A, Arendt ML, Maqbool K, Webster MT, Perloski M (2013). The genomic signature of dog domestication reveals adaptation to a starch-rich diet. Nature.

[30] Freeman LM, Michel KE (2001). Evaluation of raw food diets for dogs. J Am Vet Med Assoc.

[31] Hou JK, Abraham B, El-Serag H (2011). Dietary intake and risk of developing inflammatory bowel disease: a systematic review of the literature. Am J Gastroenterol.

[32] Alexander DD, Cushing CA (2011). Red meat and colorectal cancer: a critical summary of prospective epidemiologic studies. Obes Rev.

[33] Ijssennagger N, Belzer C, Hooiveld GJ, Dekker J, van Mil SW, Muller M (2015). Gut microbiota facilitates dietary heme-induced epithelial hyperproliferation by opening the mucus barrier in colon. Proc Natl Acad Sci U S a.

[34] O'Keefe SJ, Li JV, Lahti L, Ou J, Carbonero F, Mohammed K (2015). Fat, fibre and cancer risk in African Americans and rural Africans. Nat Commun.

[35] Song SJ, Lauber C, Costello EK, Lozupone CA, Humphrey G, Berg-Lyons D (2013). Cohabiting family members share microbiota with one another and with their dogs. Elife.

[36] Panasevich MR, Kerr KR, Dilger RN, Fahey GC, Guerin-Deremaux L, Lynch GL (2015). Modulation of the faecal microbiome of healthy adult dogs by inclusion of potato fibre in the diet. Br J Nutr.

[37] Laflamme D (1997). Development and validation of a body condition score system for dogs. Canine Pract.

[38] Thes M, Koeber N, Fritz J, Wendel F, Dillitzer N, Dobenecker B (2016). Metabolizable energy intake of client-owned adult dogs. J Anim Physiol Anim Nutr (Berl).

[39] Moxham G (2001). Waltham feces scoring system- a tool for veterinarians and pet owners. How does your pet rate?. WALTHAM®Focus.

[40] Hartviksen M, Bakke AM, Vecino JG, Ringo E, Krogdahl A (2014). Evaluation of the effect of commercially available plant and animal protein sources in diets for Atlantic salmon (*Salmo salar* L.): digestive and metabolic investigations. Fish Physiol Biochem.

[41] Anson NM, Havenaar R, Vaes W, Coulier L, Venema K, Selinheimo E (2011). Effect of bioprocessing of wheat bran in wholemeal wheat breads on the colonic SCFA production in vitro and postprandial plasma concentrations in men. Food Chem.

[42] Jouany JP, Senaud J (1983). Effect of rumen ciliates on the digestive utilization of various carbohydrate-rich diets and on the end-products formed in the rumen. II. Utilization of inulin, saccharose and lactose. Reprod Nutr Dev.

[43] Yu Y, Lee C, Kim J, Hwang S (2005). Group-specific primer and probe sets to detect methanogenic communities using quantitative real-time polymerase chain reaction. Biotechnol Bioeng.

[44] Caporaso JG, Kuczynski J, Stombaugh J, Bittinger K, Bushman FD, Costello EK (2010). QIIME allows analysis of high-throughput community sequencing data. Nat Methods.

[45] Aronesty E (2011). Command-line tools for processing biological sequencing data.

[46] Edgar RC (2013). UPARSE: highly accurate OTU sequences from microbial amplicon reads. Nat Methods.

[47] Edgar RC (2010). Search and clustering orders of magnitude faster than BLAST. Bioinformatics.

[48] DeSantis TZ, Hugenholtz P, Larsen N, Rojas M, Brodie EL, Keller K (2006). Greengenes, a chimera-checked 16S rRNA gene database and workbench compatible with ARB. Appl Environ Microbiol.

[49] Clarke K, Gorley R (2015). PRIMER v7:User Manual/Tutorial.

[50] Altschul SF, Gish W, Miller W, Myers EW, Lipman DJ (1990). Basic local alignment search tool. J Mol Biol.

[51] National center for biotechnology information (NCBI). Basic Local Alignment Search Tool (BLAST) Available from: http://blast.ncbi.nlm.nih.gov. Accessed 13 Feb 2016.

[52] Segata N, Izard J, Waldron L, Gevers D, Miropolsky L, Garrett WS (2011). Metagenomic biomarker discovery and explanation. Genome Biol.

[53] Muegge BD, Kuczynski J, Knights D, Clemente JC, Gonzalez A, Fontana L (2011). Diet drives convergence in gut microbiome functions across mammalian phylogeny and within humans. Science.

[54] De Filippo C, Cavalieri D, Di Paola M, Ramazzotti M, Poullet JB, Massart S (2010). Impact of diet in shaping gut microbiota revealed by a comparative study in children from Europe and rural Africa. Proc Natl Acad Sci U S a.

[55] Ley RE, Hamady M, Lozupone C, Turnbaugh PJ, Ramey RR, Bircher JS (2008). Evolution of mammals and their gut microbes. Science.

[56] Lopez-Siles M, Khan TM, Duncan SH, Harmsen HJ, Garcia-Gil LJ, Flint HJ (2012). Cultured representatives of two major phylogroups of human colonic Faecalibacterium prausnitzii can utilize pectin, uronic acids, and host-derived substrates for growth. Appl Environ Microbiol.

[57] Stacey M, Webb M (1947). Studies on the antibacterial properties of some basic derivatives of cholane and norcholane. Proc R Soc Med.

[58] Varela E, Manichanh C, Gallart M, Torrejon A, Borruel N, Casellas F (2013). Colonisation by Faecalibacterium prausnitzii and maintenance of clinical remission in patients with ulcerative colitis. Aliment Pharmacol Ther.

[59] Duncan SH, Hold GL, Harmsen HJ, Stewart CS, Flint HJ (2002). Growth requirements and fermentation products of Fusobacterium prausnitzii, and a proposal to reclassify it as Faecalibacterium prausnitzii gen. nov., comb. nov. Int J Syst Evol Microbiol.

[60] Duncan SH, Belenguer A, Holtrop G, Johnstone AM, Flint HJ, Lobley GE (2007). Reduced dietary intake of carbohydrates by obese subjects results in decreased concentrations of butyrate and butyrate-producing bacteria in feces. Appl Environ Microbiol.

[61] Hang I, Heilmann RM, Grutzner N, Suchodolski JS, Steiner JM, Atroshi F (2013). Impact of diets with a high content of greaves-meal protein or carbohydrates on faecal characteristics, volatile fatty acids and faecal calprotectin concentrations in healthy dogs. BMC Vet Res.

[62] Gagne JW, Wakshlag JJ, Simpson KW, Dowd SE, Latchman S, Brown DA (2013). Effects of a synbiotic on fecal quality, short-chain fatty acid concentrations, and the microbiome of healthy sled dogs. BMC Vet Res.

[63] Depauw S, Bosch G, Hesta M, Whitehouse-Tedd K, Hendriks WH, Kaandorp J (2012). Fermentation of animal components in strict carnivores: a comparative study with cheetah fecal inoculum. J Anim Sci.

[64] Yokota A, Fukiya S, Islam KB, Ooka T, Ogura Y, Hayashi T (2012). Is bile acid a determinant of the gut microbiota on a high-fat diet?. Gut Microbes.

[65] Kitahara M, Takamine F, Imamura T, Benno Y (2001). Clostridium hiranonis sp. nov., a human intestinal bacterium with bile acid 7alpha-dehydroxylating activity. Int J Syst Evol Microbiol.

[66] Ajouz H, Mukherji D, Shamseddine A (2014). Secondary bile acids: an underrecognized cause of colon cancer. World J Surg Oncol.

[67] Mentula S, Harmoinen J, Heikkila M, Westermarck E, Rautio M, Huovinen P (2005). Comparison between cultured small-intestinal and fecal microbiotas in beagle dogs. Appl Environ Microbiol.

[68] Martinez I, Perdicaro DJ, Brown AW, Hammons S, Carden TJ, Carr TP (2013). Diet-induced alterations of host cholesterol metabolism are likely to affect the gut microbiota composition in hamsters. Appl Environ Microbiol.

[69] Claus SP, Ellero SL, Berger B, Krause L, Bruttin A, Molina J (2011). Colonization-induced host-gut microbial metabolic interaction. MBio.

[70] Kostic AD, Gevers D, Pedamallu CS, Michaud M, Duke F, Earl AM (2012). Genomic analysis identifies association of Fusobacterium with colorectal carcinoma. Genome res.

[71] Castellarin M, Warren RL, Freeman JD, Dreolini L, Krzywinski M, Strauss J (2012). Fusobacterium nucleatum infection is prevalent in human colorectal carcinoma. Genome Res.

[72] Ohkusa T, Sato N, Ogihara T, Morita K, Ogawa M, Okayasu I (2002). Fusobacterium varium localized in the colonic mucosa of patients with ulcerative colitis stimulates species-specific antibody. J Gastroenterol Hepatol.

[73] van der Gaag I (1988). The histological appearance of large intestinal biopsies in dogs with clinical signs of large bowel disease. Can J Vet Res.

[74] Schäffer E (1968). Incidence and types of canine rectal carcinomas. J Small Anim Pract.

[75] Reddy BS, Weisburger JH, Wynder EL (1975). Effects of high risk and low risk diets for colon carcinogenesis on fecal microflora and steroids in man. J Nutr.

[76] Amato KR, Yeoman CJ, Cerda G, Schmitt CA, Cramer JD, Miller ME (2015). Variable responses of human and non-human primate gut microbiomes to a western diet. Microbiome.

[77] Teaford MF, Ungar PS (2000). Diet and the evolution of the earliest human ancestors. Proc Natl Acad Sci U S A.

[78] Kasiraj AC, Harmoinen J, Isaiah A, Westermarck E, Steiner JM, Spillmann T (2016). The effects of feeding and withholding food on the canine small intestinal microbiota. FEMS Microbiol Ecol.

[79] Suchodolski JS, Ruaux CG, Steiner JM, Fetz K, Williams DA (2005). Assessment of the qualitative variation in bacterial microflora among compartments of the intestinal tract of dogs by use of a molecular fingerprinting technique. Am J Vet Res.

[80] Garcia-Mazcorro JF, Lanerie DJ, Dowd SE, Paddock CG, Grutzner N, Steiner JM (2011). Effect of a multi-species synbiotic formulation on fecal bacterial microbiota of healthy cats and dogs as evaluated by pyrosequencing. FEMS Microbiol Ecol.

[81] Handl S, German AJ, Holden SL, Dowd SE, Steiner JM, Heilmann RM (2013). Faecal microbiota in lean and obese dogs. FEMS Microbiol Ecol.

[82] Wagner Mackenzie B, Waite DW, Taylor MW (2015). Evaluating variation in human gut microbiota profiles due to DNA extraction method and inter-subject differences. Front Microbiol.

[83] Wesolowska-Andersen A, Bahl MI, Carvalho V, Kristiansen K, Sicheritz-Ponten T, Gupta R (2014). Choice of bacterial DNA extraction method from fecal material influences community structure as evaluated by metagenomic analysis. Microbiome.

[84] Goodrich JK, Di Rienzi SC, Poole AC, Koren O, Walters WA, Caporaso JG (2014). Conducting a microbiome study. Cell.

[85] Guard BC, Barr JW, Reddivari L, Klemashevich C, Jayaraman A, Steiner JM (2015). Characterization of microbial dysbiosis and metabolomic changes in dogs with acute diarrhea. PLoS One.

[86] Louis P, Young P, Holtrop G, Flint HJ (2010). Diversity of human colonic butyrate-producing bacteria revealed by analysis of the butyryl-CoA:acetate CoA-transferase gene. Environ Microbiol.

[87] Suchodolski JS, Xenoulis PG, Paddock CG, Steiner JM, Jergens AE (2010). Molecular analysis of the bacterial microbiota in duodenal biopsies from dogs with idiopathic inflammatory bowel disease. Vet Microbiol.

