# Supplementary material for: A diet change from dry food to beef induces reversible changes on the faecal microbiota in healthy, adult client-owned dogs
Source: BMC Vet Res. 2017 May 30;13:147. doi: 10.1186/s12917-017-1073-9 (PMC5450340; doi:10.1186/s12917-017-1073-9)
Supplement: Supplementary file 1 — Study design, time schedule and sampling during the seven-week dietary intervention study. (DOCX 13 kb) [file 12917_2017_1073_MOESM1_ESM.docx]

**Table S1**.

Study design, time schedule, and frequency of the analysis and recordings (indicated by Xs) during the seven-week dietary intervention study

| **Weeks (No.)** |  | 1 | 2 | 3 | 4 | 5 | 6 | 7 |
| --- | --- | --- | --- | --- | --- | --- | --- | --- |
| **Diet periods^1^** |  | CD1 | | LMB | MMB | HMB | CD2 | |
| Faeces^2^ | Sequencing of bacterial DNA |  | XXX | XXX | XXX | XXX |  | XXX |
|  | Faecal consistency score |  | XXX | XXX | XXX | XXX |  | XXX |
|  | Faecal pH |  | XXX | XXX | XXX | XXX |  | XXX |
|  | Faecal water |  | XXX | XXX | XXX | XXX |  | XXX |
|  | Short chain  fatty acids |  | X | X | X | X |  | X |
| Clinical examination and weight |  | X | X | X | X | X | X | X |
| Blood samples^3^ |  |  | X |  |  | X |  |  |
| Log by owners | Faecal consistency score and diet intake | Daily | Daily | Daily | Daily | Daily | Daily | Daily |

^1^The diet periods were as follows: CD1 for week 1 and 2, during which the dogs were acclimated to the same commercial dry food diet (CD; Felleskjøpet’s Labb adult), followed by incremental substitution of CD with minced beef during the subsequent three weeks, LMB, low minced beef for week 3, MMB, moderate minced beef for week 4, and HMB, high minced beef for week 5, and finally CD2 for week 6 and 7, during which the dogs were reintroduced to the CD diet. See Table 2 for more details concerning the diets.

Dog no. 2, 8 and 9 did not contribute with samples from the following diet periods: HMB (no.2), MMB and HMB (no. 8) and CD2 (no.9).

^2^Faecal consistency score and pH were recorded in available fresh faecal samples.

^3^Blood samples were taken after HMB period, and after LMB and MMB in dog no. 8 and 2 respectively.
