# Supplementary material for: A diet change from dry food to beef induces reversible changes on the faecal microbiota in healthy, adult client-owned dogs
Source: BMC Vet Res. 2017 May 30;13:147. doi: 10.1186/s12917-017-1073-9 (PMC5450340; doi:10.1186/s12917-017-1073-9)
Supplement: Supplementary file 4 — Rarefaction analysis of V3-V4 16S rRNA gene sequences. The rarefaction curve shows observed species in samples of dogs fed different diets. (DOCX 100 kb) [file 12917_2017_1073_MOESM4_ESM.docx]

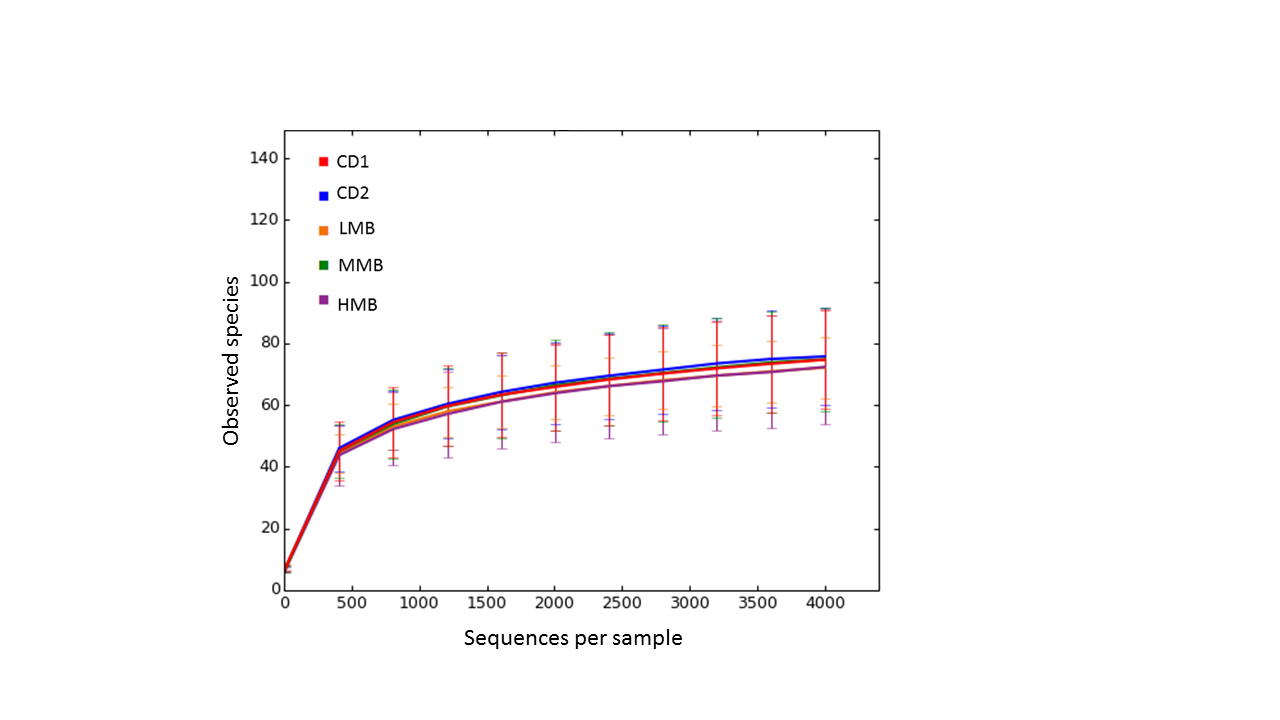


**Figure S1.** Rarefaction analysis of V3-V4 16S rRNA gene sequences. Data are from faecal samples taken following different diet periods from eleven healthy, client owned dogs during the seven-week dietary intervention study. The analysis was performed on a randomly selected subset of 4 000 sequences per sample. Each coloured curve represent a diet period: CD1 (red) for week 1 and 2, during which all dogs were acclimated to commercial dry food (CD; Felleskjøpet’s Labb adult), followed by incremental substitution of the CD diet with minced beef – LMB (yellow), low minced beef for week 3, MMB (green), moderate minced beef for week 4, and HMB (purple), high minced beef for week 5 – and finally, CD2 (blue) for week 6 and 7, during which the dogs were reintroducedto the CD diet. Average number of observed species and corresponding error bars representing standard deviation are shown for each of the diet periods.
